# The Use Of Coaching as a Novel Tool in Medical Education to Support Psychiatry Trainees in Developing Leadership Skills

**DOI:** 10.1192/bjo.2024.329

**Published:** 2024-08-01

**Authors:** Aleksandra Szczap

**Affiliations:** Tavistock and Portman NHS Foundation Trust, London, United Kingdom. Anglia Ruskin University, Cambridge, United Kingdom. Fellowship of Higher Education Academy, London, United Kingdom

## Abstract

**Aims:**

In recent years, there has been an increasing recognition of the importance of leadership skills to doctors. However, these skills are not usually formally taught or assessed. This study aims to evaluate the impact of using coaching as a novel technique to support Psychiatry trainees in developing leadership skills.

**Methods:**

This poster summarises a primary research study which evaluated an educational intervention using a mixed-method approach.

All Higher Psychiatry Trainees at the Tavistock and Portman NHS Foundation Trusts were given an opportunity to enrol on the *Coaching for Consultant Psychiatrist Leadership Pilot Programme.* Those who enrolled (coachees) were offered five 90-minute one-to-one sessions with Psychiatry consultants (coaches) who received training in *Coaching for Leadership*. Coachees completed a self-assessment questionnaire examining their views on their own leadership skills before and after completing the programme. Questions used were adopted from the High-Performance People Skills questionnaire (HPPS) – a tool used to collect 360° feedback as part of leadership training. Coachees and coaches also attended separate focus groups to discuss their subjective experiences of receiving and providing coaching. Thematic analysis was carried out.

**Results:**

Eight coaches and seven coachees participated in the study. Most HPPS self-assessment scores post-intervention have increased after completion of the course. Overall coachees' satisfaction with their leadership skills increased from 3.4/5.0 to 4.0/5.0 after completing the programme.

Focus groups yielded rich qualitative data. The themes identified were: a broad range of reasons for Trainees and Consultants to join the programme, positive impact on coaches’ coaching skills, positive impact on coachees’ leadership skills and ideas on how to improve the programme to inform future curriculum design or improve implementation of any educational interventions.

**Conclusion:**

Psychiatry Trainees’ self-perception of leadership skills can improve after receiving coaching. They find coaching helpful due to its individualised nature and the fact that it promotes the application of skills in real-life settings. Coaches also benefit by gaining new skills in coaching and leadership. Coachees and coaches share similar motivations for joining, including the desire to learn new skills, support others and be supported. More research is needed to evaluate the practical aspects of delivering coaching for leadership programmes as part of speciality training, but its potential is promising.

